# Behavioral and Neurobiological Correlates of Resilience in the Hindlimb Unloading Mouse Model: A Longitudinal Ethogram with Neurotrophin Profile

**DOI:** 10.3390/life16010137

**Published:** 2026-01-15

**Authors:** Arianna Racca, Patrizia Pignataro, Roberta Zerlotin, Graziana Esposito, Bijorn Omar Balzamino, Alessandra Micera, Maria Grano, Daniela Santucci

**Affiliations:** 1 Center for Behavioral Sciences and Mental Health, Istituto Superiore di Sanità, 00161 Rome, Italy; 2 Department of Translational Biomedicine and Neuroscience—DiBraiN, University of Bari, 70124 Bari, Italy; pa.pignataro@gmail.com (P.P.); maria.grano@uniba.it (M.G.); 3 Research and Development Laboratory for Biochemical, Molecular and Cellular Applications in Ophthalmological Science, IRCCS—Fondazione Bietti, 00184 Rome, Italy; graziana.esposito@fondazionebietti.it (G.E.); bijorn.balzamino@fondazionebietti.it (B.O.B.);

**Keywords:** ethogram, neurotrophins, hindlimb unloading model, animal welfare, mouse

## Abstract

Among ground-based paradigms used to reproduce altered gravity exposure, the hindlimb unloading (HU) model is widely employed to simulate microgravity conditions by removing gravitational loading from the hindlimbs. Despite its extensive use, behavioral adjustments during suspension remain poorly characterized, although they may provide valuable indicators of animal welfare and individual susceptibility. Here, we comprehensively characterized the behavioral profile of mice during and after HU using a dedicated ethogram, with the aim of identifying behavioral markers associated with individual coping strategies. Several exploratory and postural behaviors showed marked time-dependent modulation, with baseline exploratory activity predicting a more adaptive behavioral trajectory during suspension, possibly indicative of greater resilience. In parallel, brain levels of the neurotrophins NGF and BDNF were measured to explore their relationship with behavioral outcomes. Although no significant group differences were detected, suspended mice displayed a progressive reduction in both neurotrophins over time, which paralleled behavioral adaptation. Together, these findings indicate that specific exploratory behaviors represent reliable predictors of resilience to HU, while NGF and BDNF may reflect ongoing neuroplastic processes associated with prolonged suspension.

## 1. Introduction

Animals have contributed greatly to the current database of knowledge in space biology [1]. After the first living creature, the dog Laika, was launched into space (1966), a large variety of vertebrate and invertebrate species have been selected to carry out experiments in zero gravity [1]. Animals are sent into orbit to proactively predict health problems in humans and they are routinely exposed to un-physiological gravity on Earth to understand physiological adaptations in such an environment and investigate possible countermeasures to reduce animal and human suffering in space.

Among ground-based models used to reproduce the effects of simulated microgravity conditions in rodents [2,3], the hindlimb unloading model (HU) is a well-established paradigm largely employed to reproduce the effects of simulated microgravity conditions on Earth [2,3]. It selectively consists of unloading the rat or mouse hindquarters by suspending the animal by its tail, maintaining an unloading angle of 30° in order to provide normal weight bearing on the forelimbs and reproduce reliable physiological responses found in microgravity. Several studies reported that many physiological changes observed in the HU model are similar to those encountered in space. The HU model induces disuse osteopenia [4,5], a cephalad-fluid shift and cerebral blood flow [6], as occurs in astronauts in space [7]. Both the HU model and space flight limit forelimb and hindlimb movements, inducing a reduction in muscle mass and reproducing muscle atrophy in the load-bearing muscles [8]. Analogously to the effects reported in a space environment, the immune system is compromised, and some metabolic changes as well as cardiovascular effects confirm the translational value of the model [6,9,10,11]. More recently, several studies attempted to demonstrate the reliability of this model as a tool to also investigate the physiology of the nervous system, with particular regard to the effects on brain neuroplasticity, gene expression, or the electrophysiological currents of neuronal cells [3,12,13,14,15].

Although it is widely recognized as a valid paradigm for simulating ground-based microgravity, little is known about the neurobehavioral adaptive responses of animals subjected to this procedure—or about the wide range of individual coping strategies they exhibit in response to this unnatural posture.

Indeed, results obtained from recent decades demonstrate that the complexities of the physiological adaptations to changing environmental conditions, such as un-physiological gravity, require a shift in focus to an integrated approach in which a wider range of functional, structural and behavioral information converges [16,17]. In particular, the brain structure and the mind comprise complex systems characterized by mechanisms of recurrence, feedback and interconnectivity that make it difficult to reduce the individual response to genetic or molecular biomarkers [18,19]. Moreover, the scientific results collected over many years of space exploration have demonstrated that individuals respond differently to the condition of microgravity, and to understand the origin of this variability in such vulnerability, it is necessary to elucidate the mechanisms leading to the individual differences [20,21].

In this context, a detailed behavioral analysis represents a tool to investigate the initial and adaptive response to simulated microgravity conditions. Although neuronal circuits play a fundamental role in the investigation of mechanisms of adaptive behaviors, it has been largely demonstrated that the behavioral endpoints depend above all on interactions among the nervous system, body and environmental challenges [18,22,23], eliciting valid indicators/elements of tolerability to changing environmental challenges [24]. In the HU model, a classical stress paradigm, one of the most evident weaknesses specifically regards the inconsistencies in the study of stress-related responses, which requires detailed knowledge about animals’ individual reactions to such a procedure; behavioral outcomes depend not only on the type of stressor but also on the coping strategy that characterizes each experimental subject. Investigating such individual variation could explain why some conditions that are well tolerated by some individuals may be detrimental to others, this being a key element for improving animal welfare, screening resilience to altered gravitational environments and developing appropriate countermeasures. In experiments using the hindlimb unloading (HU) model, social isolation plays a pivotal role in the interpretation of results. It is well established that socially isolated mice display distinct behavioral and neurobiological profiles [25], including exacerbated depressive-like behaviors and altered immune function [26]. In line with this, Naumova and colleagues (2023) demonstrated that the detrimental effects of the HU procedure on stress-related hippocampal markers are primarily driven by social isolation rather than by unloading per se [27]. Conversely, previous work reported that HU increased the adrenal gland mass in socially housed mice compared with socially isolated animals, without concomitant differences in musculoskeletal outcomes [15].

Within this framework, investigating individual behavioral responses to stressful conditions becomes essential for identifying indicators of susceptibility to the HU model. To date, only a limited number of studies have addressed individual variability in response to hindlimb unloading. For instance, Friedman and colleagues examined genetic predictors of vulnerability by comparing skeletal responses to HU across different mouse strains, revealing strain-specific effects on bone metabolism genes and suggesting a role of genetic background in shaping susceptibility [28].

However, individual responses to HU likely emerge from the interaction of multiple and complex variables that act synergistically to shape behavioral and physiological outcomes. In this context, the development of a detailed ethogram capable of capturing spontaneous behavioral reactions during and after suspension represents a valuable approach to identifying individual vulnerability, monitoring animal welfare, and defining behavioral indicators of susceptibility throughout and following the unloading period.

The ethological approach also requires a comprehensive evaluation of individual profiles for ensuring proper care and behavioral assessment during the experimental study [29]. This underlines the need for defining behavioral indicators that can be used to evaluate behavioral adjustments in animals subjected to the HU procedure, as well as for enhancing the procedure to reduce psychophysical suffering.

The aim of the present study was to analyze the neurobehavioral profile of mice subjected to the HU paradigm in order to identify behavioral biomarkers of susceptibility to simulated microgravity conditions. In particular, mice were exposed to different durations of suspension procedures and their behavioral repertoire was evaluated during and after the end of the suspension. A specific ethogram was defined as a tool to both monitor the behavioral profile of the animals during the suspension and compare the adaptive outcome with post-suspension data. Furthermore, since changes in neurotrophin levels in the central nervous system have been largely reported upon exposure to gravitational stimuli and stressful experiences [30,31,32,33] in relation to their role as modulators of synaptic plasticity, it is also considered of interest to investigate whether the HU procedure has an impact on such neurobiological determinants.

## 2. Materials and Methods

This study was designed and reported in accordance with the ARRIVE guidelines [34]. Animals were randomly allocated to experimental groups. All procedures were approved by the Institutional Animal Care and Use Committee and conducted in compliance with European and national legislation. Sample sizes were determined on the basis of previous studies, using the minimum number of animals required given the severity of the hindlimb unloading procedure. All animals meeting the inclusion criteria, except one, were included in the analyses, and no data were excluded. Behavioral assessments were conducted from video recordings using standardized ethological scoring methods. Key experimental conditions, including housing, suspension protocol and outcome measures, are reported to ensure transparency and reproducibility.

### 2.1. Animals

Animal procedures were performed in accordance with the European and National legislation (EU 63/2010, DL 26/2014), and protocols were approved by Institutional Animal Care and Use Committee (authorization n: N. 12/2022-PR). Upon arrival at the laboratory of University of Bari, C57BL6 mice animals were housed under standard conditions in an air-conditioned room (temperature 21 ± 1 °C, relative humidity 60 ± 10%; 12 h–12 h light/dark cycle) in 42 cm × 27 cm × 14 cm Plexiglas cages with a metal top and sawdust as bedding. Pellet food and tap water were provided ad libitum.

### 2.2. Hindlimb Unloading Protocol

After a period of adaptation (one week), adult male and female C57BL6 mice (two months old, n = 44) were weighed and randomly assigned to six groups: three groups of normal weight-bearing control mice, one group for each experimental group of suspended mice (CTR groups: one week (CTR1, n = 6: 2 females and 4 males), two weeks (CTR2, n = 7: 4 females and 3 males), and three weeks (CTR3, n = 7: 2 females and 5 males) and three groups of hindlimb-suspended mice (HU groups: one week of suspension WK1, n = 7: 3 females and 4 males, one group for two weeks WK2, n = 7: 4 females and 3 males, one group for three weeks WK3, n = 10: 6 females and 4 males). All experimental subjects (CTR/HU) were maintained in social isolation from the beginning of the experiment to euthanasia in order to evaluate the behavioral effects associated with the suspension procedure, avoiding the confounding variable induced by social housing. The suspension procedure was realized according to recommendations by Wronski and Morey-Holton [35]. The hindlimbs of the HU group were elevated to a spinal orientation of 30° above horizontal to simulate a shift in body fluids similar to that experienced during space flight. The elevation was adjusted in order to avoid any contact of the hindlimbs with the cage floor and the forelimbs were free to ambulate in the entire range of the cage, and the mice had continuous access to food and water. To maintain the animals suspended, a strip of orthopedic tape, attached to a plastic suspension bar, was applied to the sides of the tail, after spraying and letting the benzoin tincture dry on the tail skin to protect against irritation from the adhesive tape. Each mouse was then attached via the plastic suspension bar to a pulley system mounted on top of the cage.

### 2.3. Experimental Design

To assess the neurobehavioral profile of the animals and identify potential predictive behavioral markers of vulnerability or resilience to simulated microgravity, their behavior was evaluated both during and after the suspension procedure. Specifically, each subject’s behavioral repertoire was monitored and characterized across the weeks of suspension, and their overall behavioral profile was assessed at the end of the procedure by examining spontaneous activity in the home cage and responses to a novel object. Behavioral assessments were conducted by an observer blinded to the weeks of suspension and sex of the mice. Following the behavioral testing, animals were weighed and sacrificed, and brain samples were collected for the quantification of NGF and BDNF levels.

### 2.4. HU Ethogram

**Figure 1:**
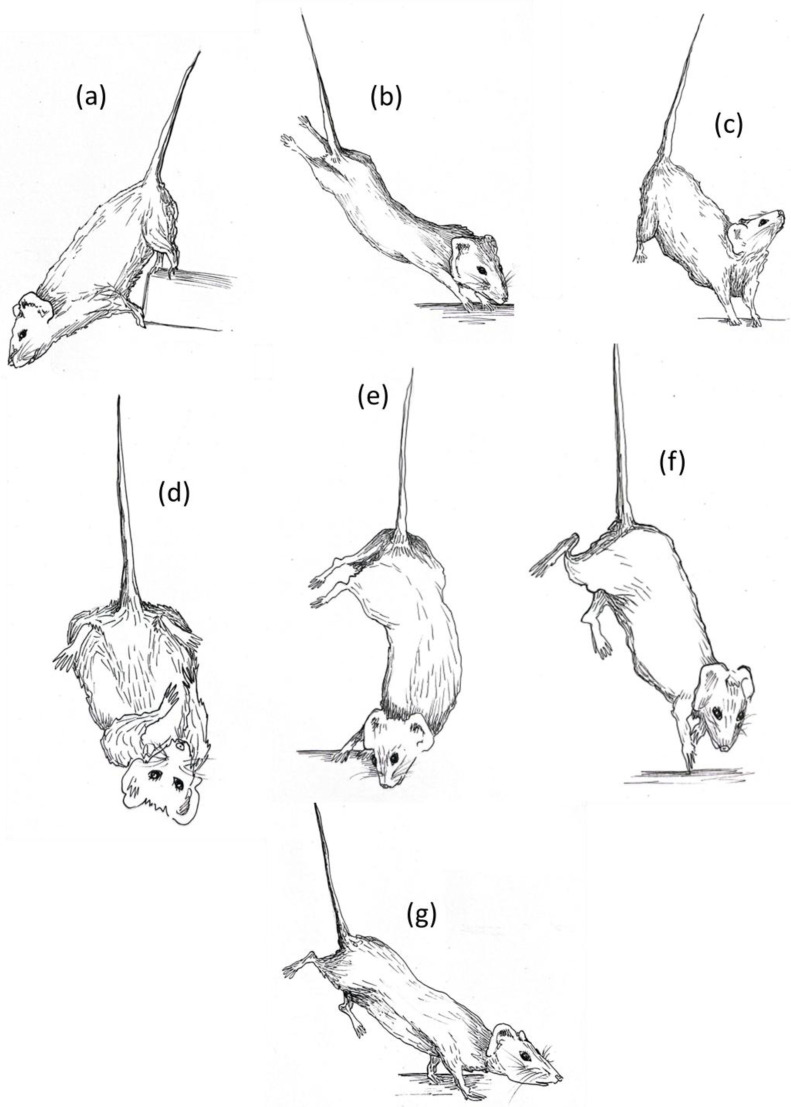
HU ethogram: (**a**) *forelimbs hanging*, (**b**) *fully extended hindlimbs*, (**c**) *vertical head raising*, (**d**) *self-grooming*, (**e**) *alternative extended hindlimbs*, (**f**) *running*, (**g**) *exploring*. The illustrations were made by Ilaria Racca.

Each experimental subject was videorecorded for 10 min across the three weeks of suspension to monitor the behavioral profile during the entire suspension period. According to the temporal dimension of each experimental group, mice were videorecorded and observed at the precise moment at which they were suspended (“just suspended”), and after one day (day1) and one, two, and three weeks (week1, week2, week3) of suspension. After several behavioral observations, a detailed ethogram was defined and the behavioral profile of each animals was scored using dedicated ethological software (“The Observer^®^ XT 15.0“ by Noldus). The drawings in this paper are taken directly from these videos (Figure 1).

### 2.5. Spontaneous Behavior and Novel Object Test

To assess locomotor activity and the approach to novelty, the animals were tested in a novel object test after the end of the suspension procedure. Each experimental subject was placed randomly at the center of a plexiglass cage with sawdust (42 cm × 27 cm × 14 cm) and videorecorded for 7 min. In particular, after 5 min of spontaneous activity evaluation, a stimulus object (a glass marble) was placed at the center of the half-cage, and latency, number and duration of *sniffing contacts* with the object were recorded for 5 min. The position of the marble between the two halves of the cage was randomized for each animal. Videos were scored using “The Observer^®^ XT 15.0” software, and frequency, duration and latency of the following behavioral items were also recorded: *exploring* (locomotor activity in the cage), *wall rearing* (vertical exploration with hindpaws on the floor and forepaws on the wall of the arena), *rearing* (vertical exploration with hindpaws on the floor and forepaws in the open), *grooming* (self-explanatory), *inactivity* (lying flat or standing still in total absence of movement), and *digging* (digging up the sawdust). New, clean cages were used for each animal. The recording session took place between 1.00 and 5.00 pm in an experimental room maintained at the same temperature and humidity conditions as the housing one.

### 2.6. Brain Preparation Before ELISA

Brain samples, collected randomly for each experimental subject, were Trypsin-EDTA-harvested and pellets were homogenized by ultra-sonication (Sonics, Newtown, CT, USA) in RIPA buffer (50 mM Tris-Cl, pH7.5; 150 mM NaCl; 5 mM EDTA; 1% Triton X-100; 0.1% SDS; 0.5% DOC (Sodium deoxycholate; 1 mM PMSF; 1 μg/mL leupeptin). Clear supernatants (4 °C/13,000 rpm, 20 min) were collected and used for quantification of neurotrophin levels.

### 2.7. Quantification of Neurotrophin Levels Using ELISA on Brain Samples

For NGF and BDNF quantifications, double-sandwich ELISA assays were used. In particular, 96-well Maxisorp plates (Nunc, Roskilde, Denmark) were pre-coated with the specific capture antibodies (0.4 μg/mL; R&D) overnight (4 °C). Brain samples were diluted 1:2 in assay diluent (from the kit; R&D) supplemented with 1× protease inhibitor cocktail (Pierce—Thermo Fisher Scientific Inc. Waltham, MA, USA) and loaded in parallel with the standard curve (0.32–2000 pg/mL protein; R&D). Overnight incubation with the sample was performed, and subsequently, addition of the specific detection secondary antibodies (0.15 μg/mL; R&D) and streptavidin (1:200; R&D) was carried out. Specific binding was developed by using the ready-to-use TMB substrate and Stop solution (R&D). The colorimetric signals (Optic Density, OD) were acquired at λ 450–570 nm by using the Sunrise plate reader (Tecan Group Ltd., Männedorf, Switzerland). The related target values (pg/mL) were produced using a third-grade polynomial standard. The absence of cross-reactivity with other neurotrophins was declared by the manufacturer for both assays.

### 2.8. Real-Time RT-PCR: Transcriptomic Analysis on Optic Nerves

The analysis was performed on optic nerve heads, collected before the brain tissues, and extracted/amplified according to a standardized procedure carried out for a few selected mediators (BDNF, NGF, p75^NTR^, iNOS, Bcl2, Bax and GFAP). Target gene expressions were provided by the software (row data) according to the 2–ddCt formula (ddCt = dCt sample − dCt calibrator). The single-target gene expressions (fold changes [FCs]) were expressed on the log2 scale, as directly provided by Illumina software (version 3.1.7), with respect to the control group (normal values). REST-384 (2006) software (version 2) was also used to estimate changes in transcript expression, as calculated with respect to the reference genes (H3, GAPDH, 2MG).

### 2.9. Statistical Analysis

Continuous variables are presented as means and standard errors of means (S.E.M.). The normality of the distribution was tested with the Shapiro–Wilk test.

Behavioral parameters recorded across the three weeks of suspension were analyzed using R software (version 4.5.2). The duration and frequency of the behavioral data were analyzed using linear mixed-effects models (LMMs) to account for the repeated-measures design.

For each behavioral variable, models included sex, period of suspension (just suspended, day1, week1, week2, week3), and their interaction as fixed effects, with subject identity (ID) included as a random intercept. For behaviors showing no sex-related effects, estimated marginal means were computed by pooling data across sexes.

Models were fitted using REML (*lme4* package, version 1.1-38). Fixed effects were tested using Type III ANOVA with Satterthwaite’s approximation (*lmerTest* package, version 3.1-3). When significant effects were detected, post hoc Tukey-adjusted comparisons were performed using estimated marginal means (*emmeans* package, version 2.0.1), with degrees of freedom estimated using the Kenward–Roger method.

Data collected after the end of the suspension period were analyzed by one-, two- or three-way ANOVA, with week of suspension (three levels: week1 vs. week2 vs. week3), treatment (two levels: control vs. hindlimb unloading) or sex (two levels: male vs. female) as between factors.

When the interaction between treatment and another independent variable was significant, post hoc comparisons were performed using a Tukey HSD test. Multiple comparisons were applied to logical sets of means according to the specific objectives of the work.

When the normality was not met, the non-parametric Mann–Whitney test for independent samples was used.

Latency data, which had no normal distribution, were analyzed with the Mann–Whitney nonparametric test.

Pearson correlations were applied to associate behavioral profiles or neurobiological data during and after suspension.

Comparisons were performed by using Statview (version 5.0.1) software and statistical significance was set a *p* < 0.05.

## 3. Results

### 3.1. Suspended Animals Exhibited Peculiar Behavioral Adaptations During Suspension

Several behavioral elements and subtle deviations from the typical species-specific ethogram were observed in mice during the suspension procedure. Notably, postures were grouped into behavioral categories that appeared to share similar motivational underpinnings. The specific behavioral elements included in the hindlimb unloading (HU) ethogram are illustrated in Figure 1 and listed below.

Explorative behaviors:

- *Exploring* is performed when the animal changes the position of its body moving the forelimbs. It is usually related to a simple direction of attention towards an external stimulus, to heading towards food/water or to moving around the cage. *Sniffing* is often associated with this behavior.
- *Fully extended hindlimbs* emerges when the animal stretches its hindlimbs upward in order to bring its head towards the base of the cage. When mouse is stretching, there is usually close contact between its head or forelimbs and some parts of the cage (walls, sawdust, food, water). For these reasons, it is very often performed simultaneously with the *sniffing* behavior.
- *Forelimbs hanging* is evidenced when the animal grabs a component of the cage with the forelimbs or the mouth. In general, it is performed rarely and could represent a behavioral strategy to reach the top of the cage and try to escape from the suspension position.
- *Vertical head* raising is observed when the animal raises its head upward. In general, the animal is in resting position and counteracts the gravity force, raising its head in order to explore the environment on the top of the cage. It could be an explorative index or a self-maintenance behavior associated with the need to change the orientation of the head and move the anterior part of the body upward. It is often performed simultaneously with *sniffing* behavior and it could be preceded or followed by *immobility* behavior.
- *Immobility* behavior is defined as a posture where the mouse hangs without engaging in any activity and keeps the forelimbs at the base of cage.

Balancing behaviors:

- *Running* is performed when the animal moves its forelimbs and/or hindlimbs frantically in order find a balanced position again. It is defined as “balancing behavior” because it usually emerges when the animal has just lost balance due to the movement of the body and tries to find a permanent resting position again.
- *Alternative extended hindlimbs* is evidenced when the animal rotates its body, moving the hindlimbs unilaterally in the opposite direction guided by the head and forelimbs. This specific posture allows the animal to maintain a vertical position and remain with the forelimbs on the base of the cage.

Maintenance behaviors:

- These behaviors usually occur in sequence. During *self-grooming*, the animal brings the head towards the belly and begins to wash its face with the licked forelimbs (*face washing behavior*), drawing them over the head from just behind the ears. Very often it is accompanied by *vertical scratching* behavior where the mouse maintains its forelimbs at the base of the cage and scratches the ear with the hindlimbs.

The behavioral analysis across the weeks evidenced a critical time point in the coping strategy during the suspension procedure.

**Figure 2:**
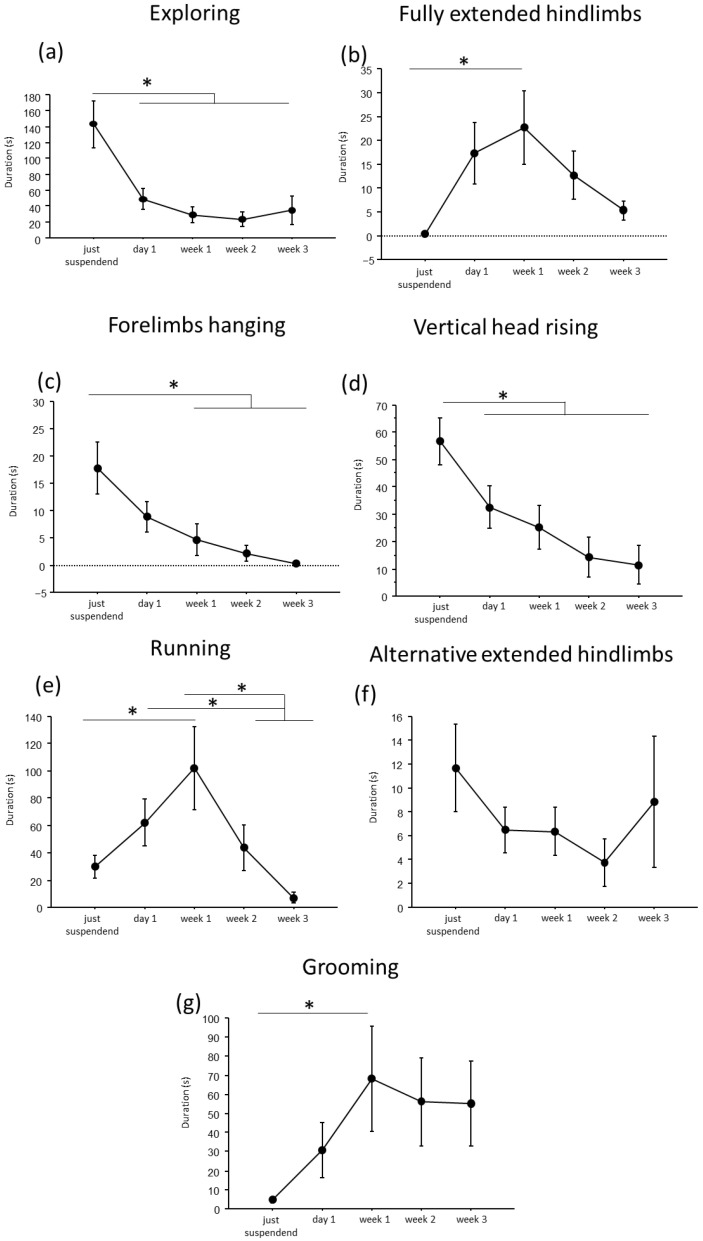
Behavioral analysis during suspension procedure. During the first day of suspension, a reduction in duration of *exploring* (**a**), *forelimbs hanging* (**c**), *vertical head raising* (**d**), and a trend up to the week2 in *alternative extended hindlimbs* (**f**), was evidenced, while an increase was observed in the durations of *fully extended hindlimbs* (**b**), *running* (**e**) and *grooming* (**g**) behaviors. Data from males and females are pooled and shown as estimated marginal means from a linear mixed-effects model with *period of suspension* as a fixed effect and *subject* as a random effect. Estimates are based on Tukey-adjusted pairwise comparisons, with degrees of freedom computed using the Satterthwaite method. * Statistically significant at *p* < 0.05. Just suspended = 8, day1 = 8, week1 = 8, week2 = 8, week3 = 8.

**Figure 3:**
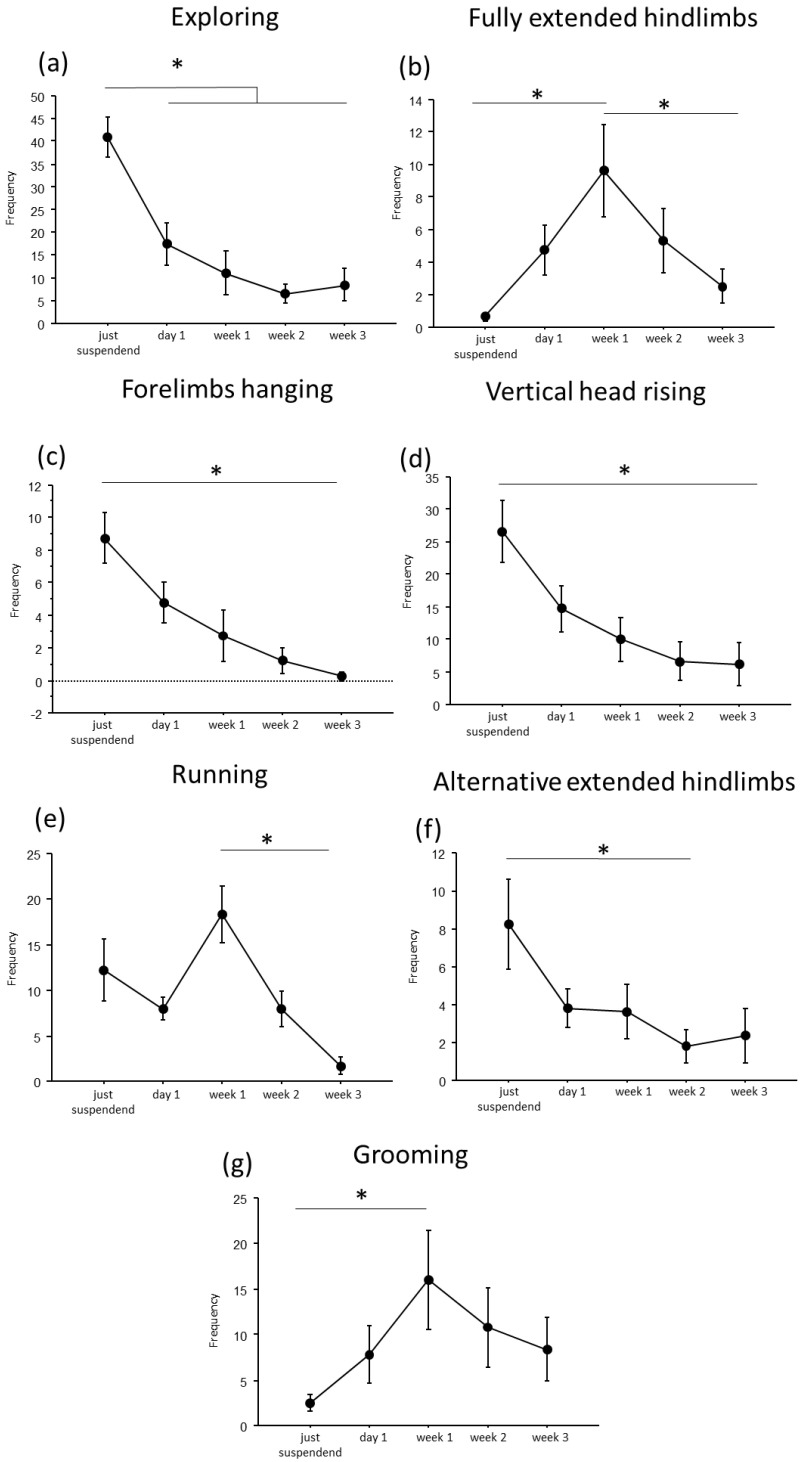
Behavioral analysis during suspension procedure. During the first day of suspension, a reduction in frequency of *exploring* (**a**), *forelimbs hanging* (**c**), *vertical head raising* (**d**), and *alternative extended hindlimbs* (**f**), was evidenced, while an increase was observed in the frequency of *fully extended hindlimbs* (**b**), *running* (**e**) and *grooming* (**g**) behaviors. Data from males and females are pooled and shown as estimated marginal means from a linear mixed-effects model with *period of suspension* as a fixed effect and *subject* as a random effect. Error bars represent 95% confidence intervals. Estimates are based on Tukey-adjusted pairwise comparisons, with degrees of freedom computed using the Satterthwaite method. * Statistically significant at *p* < 0.05. Just suspended = 8, day1 = 8, week1 = 8, week2 = 8, week3 = 8.

A convergent critical time point of adaptive behavioral trends at day1 of suspension was evident, during which a reduction in *explorative behaviors*, except for the *fully extended hindlimbs* behavior, and a concomitant increase in *running* and *grooming* behaviors were observed (Figure 2 and Figure 3).

In particular, throughout the weeks, suspended mice showed a decrease in duration and frequency of *explorative behaviors*, such as *Exploring* (duration: F_(4,19.972)_ = 9.7435, *p* < 0.001; frequency: F_(4,24.09)_ = 16.80; see Figure 2 and Table 1 for post hoc comparison), *forelimbs hanging* (duration: F_(4,25.08)_ = 7.32, *p* < 0.001; frequency: F_(4,25.47)_ = 9.22, *p* < 0.001), and *vertical head raising* (duration: F_(4,23.44)_ = 10.47, *p* < 0.001; frequency: F_(4,22.61)_ = 10.20, *p* < 0.001). Interestingly, the opposite trend was observed in *fully extended hindlimbs* behavior (duration: F_(4,25.06)_ = 4.01, *p* = 0.012; frequency: F_(4,24.85)_ = 7.53, *p* < 0.001) with a significant increase in duration and frequency during week1 compared to just-suspended mice.

Concerning *balancing behaviors*, an effect of the suspension was evident in the duration and frequency of *running* (duration: F_(4,23.67)_ = 9.21, *p* < 0.001; frequency: F_(4,23.64)_ = 11.98, *p* < 0.001) and in the frequency of *alternative extended hindlimbs* (F_(4,21.27)_ = 3.22, *p* = 0.033). Specifically (see Table 1 for post hoc comparisons), a significant increase during week1 was observed in the duration and frequency of *running* behavior, while a significant reduction was observed in the frequency of *alternative extended hindlimbs* between just suspended and week1 and week2.

Regarding the analysis of *maintenance behaviors*, a progressive increase both in duration and frequency (F_(4, 23.77)_ = 3.55, *p* = 0.021) of *self-grooming* was observed from week1.

Concerning sex-related differences (see Figure S1 and Table S1 in Supplementary Materials), a significant increase in *balancing behaviors* was observed in females at week1 for *running behavior* (sex × period of suspension: duration: F_(4, 23.67)_ = 3.46, *p* = 0.023; frequency: F_(4, 23.64)_ = 3.95, *p* = 0.014), while males showed a significant increase in both duration and frequency of *vertical head raising behavior* (sex × period of suspension: duration: F_(4, 23)_ = 3.95, *p* = 0.01; frequency: F_(4, 22.6)_ = 4.9458, *p* = 0.005) during the first periods of suspension.

### 3.2. The Suspension Procedure Affects Animal Body Weight per Se, Regardless of the Duration of Exposure

**Figure 4:**
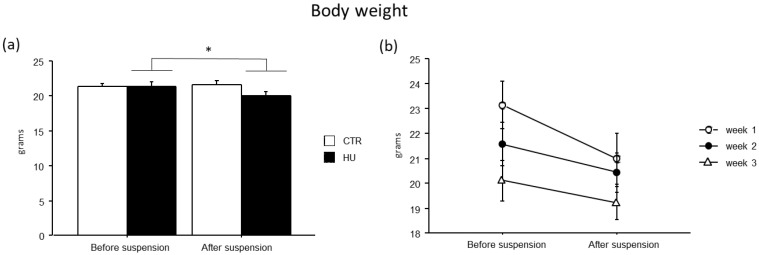
Body weight before and after suspension. A modest reduction in body weight was observed in suspended mice (**a**), without significant differences among the experimental groups (**b**). Data are shown as means ± S.E.M. * Statistically significant at *p* < 0.05 using repeated-measure ANOVA followed by post hoc Tukey HSD test. CTR before suspension = 20, CTR after suspension = 20, HU before suspension = 24 (week1 = 7, week2 = 7, week3 = 10), HU after suspension = 24 (week1 = 7, week2 = 7, week3 = 10).

All suspended animals showed a modest reduction in body weight (F_(1,43)_ = 9.892, *p* = 0.003, *p* < 0.05 after post hoc comparisons), while no significant differences were found among the experimental groups (Figure 4).

### 3.3. The Duration of Suspension Has a Significant Impact on the Explorative and Emotional Profiles of Mice

**Figure 5:**
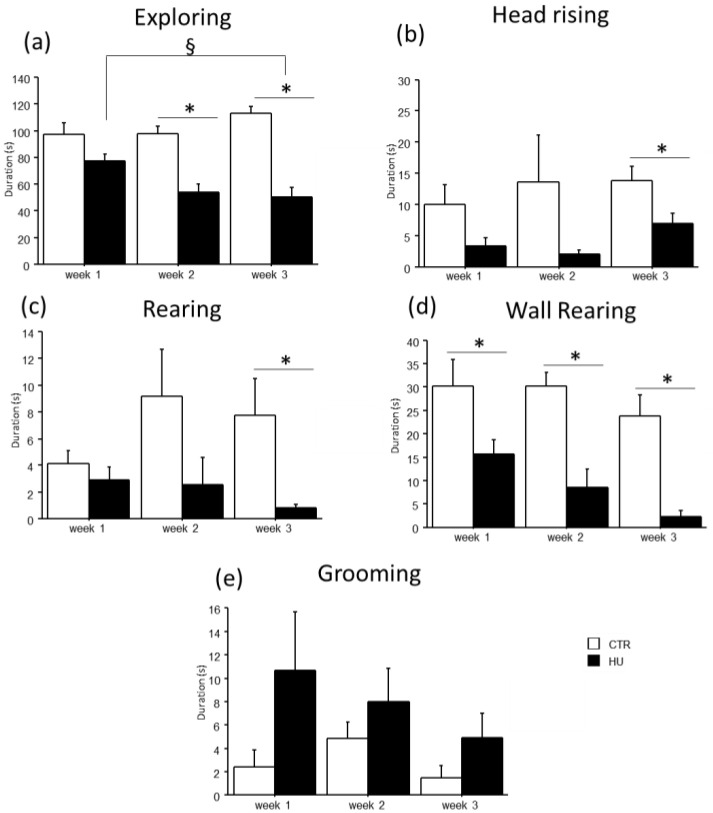
Behavioral effects after suspension procedure. A general reduction in the durations of explorative behaviors and vertical movements was observed after one (**b**,**d**), two (**a**,**d**), and three weeks (**a**–**d**), while a tended increase in *grooming* behavior was observed in suspended mice (**e**). Data are shown as means ± S.E.M. * Statistically significant at *p* < 0.05 using one-way ANOVA or Mann–Whitney test (**b**–**d**), § Statistically significant at *p* < 0.05 using two-way ANOVA post hoc by post hoc Tukey HSD test. CTR week1 = 6, CTR week2 = 7, CTR week3 = 7, HU week1 = 7, HU week2 = 7, HU week3 = 10.

**Figure 6:**
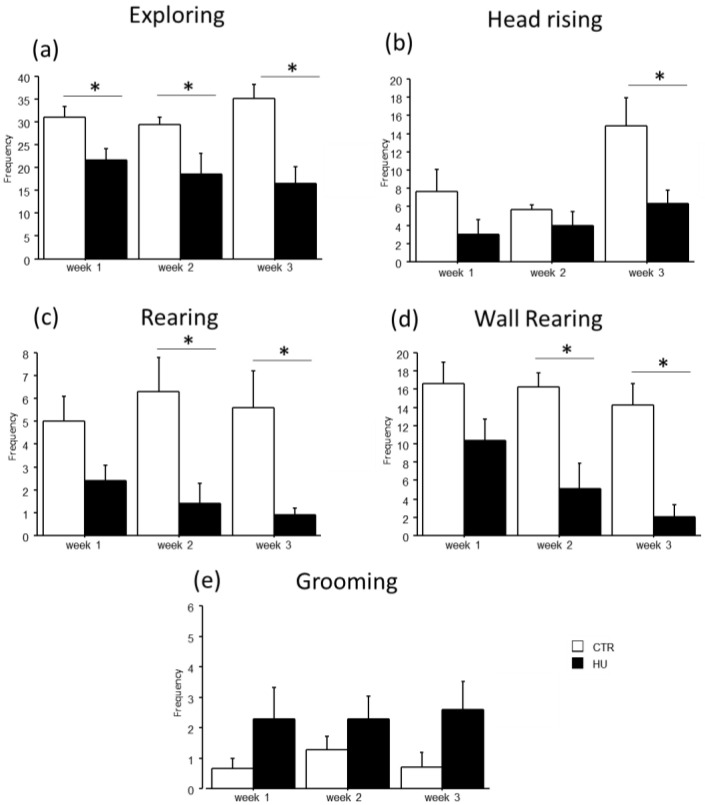
Behavioral effects after suspension procedure. A general reduction in the frequencies of explorative behaviors and vertical movements was observed after one (**b**), two (**a**,**c**,**d**), and three weeks (**a**–**d**), while a tended increase in *grooming* behavior was observed in suspended mice (**e**). Data are shown as means ± S.E.M. * Statistically significant at *p* < 0.05 using one-way ANOVA or Mann–Whitney test (**c**). CTR week1 = 6, CTR week2 = 7, CTR week3 = 7, HU week1 = 7, HU week2 = 7, HU week3 = 10.

In the novel object test, the duration of some explorative behaviors was reduced in suspended mice when compared to relative controls (Figure 5 and Figure 6). In particular, a significant reduction in *Exploring* behavior was observed after week1 (frequency: F_(1,11)_ = 7.484, *p* = 0.0194 between mice suspended for one week and relative controls), week2 (duration: F_(1,12)_ = 27.745, *p* = 0.0002 between mice suspended for two weeks and relative controls; frequency: F_(1,12)_ = 4.944, *p* = 0.0462 between mice suspended for two weeks and relative controls) and week3 (duration: F_(1,15)_ = 39.668, *p* < 0.0001 between mice suspended for three weeks and relative controls; frequency: F_(1,15)_ = 13.372, *p* = 0.0023 between mice suspended for three weeks and relative controls). A significant reduction in *rearing* behavior was observed after week2 (frequency: U = 44.500, *p* = 0.0106 between mice suspended for two weeks and relative controls) and week3 (duration: U = 65.000, *p* = 0.0034 between mice suspended for three weeks and relative controls; frequency: U = 55.500, *p* = 0.0057 between mice suspended for three weeks and relative controls), as well as *wall rearing* after week1 (duration: U = 34.000, *p* = 0.06 between mice suspended for one week and relative controls), week2 (duration: U = 46.000, *p* = 0.006 between mice suspended for two weeks and relative controls; frequency: F_(1,12)_ = 12.291, *p* = 0.0043 between mice suspended for two weeks and relative controls) and week3 (duration: U = 66.000, *p* = 0.0025 between mice suspended for three weeks and relative controls; frequency: F_(1,15)_ = 23.618, *p* = 0.0002 between mice suspended for three weeks and relative controls). Finally, a significant reduction in *head raising* behavior was observed after week3 (duration: U = 56.000, *p* = 0.04 between mice suspended for three weeks and relative controls; frequency: F_(1,15)_ = 7.472, *p* = 0.0154 between mice suspended for three weeks and relative controls). Although no significant differences were revealed, it is interesting to note that suspended mice tended to increase the duration and frequency of *grooming* behavior compared to control mice. Concerning the interaction between week and treatment, post hoc analysis revealed a decrease in the duration of *Exploring* behavior between mice suspended for three weeks and those suspended for one week (duration: F_(2,38)_ = 5.306, *p* = 0.0093; *p* < 0.05 after post hoc comparison).

**Figure 7:**
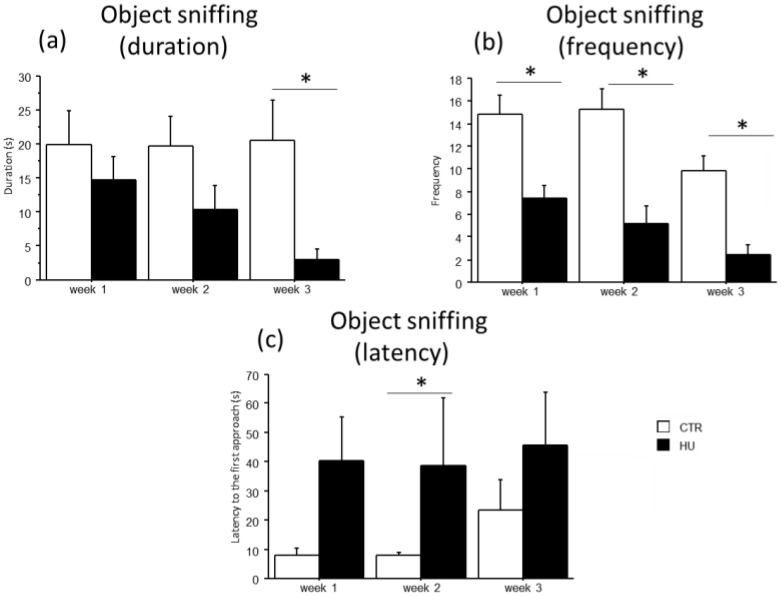
Effect of suspension on the emotional profile of mice. Suspended mice showed a general reduction in the *object sniffing* behavior (**a**,**b**) with a concomitant increase in the latency to the first approach to the novel object (**c**). * Statistically significant at *p* < 0.05 using one-way ANOVA (**b**) and Mann–Whitney test (**a**,**c**). CTR week1 = 6, CTR week2 = 7, CTR week3 = 7, HU week1 = 7, HU week2 = 7, HU week3 = 10.

A general decrease in *object sniffing* in suspended mice and a concomitant increase in the latency to the first approach to the *novel object* were observed (Figure 7). Specifically, an effect of treatment was found for the duration and frequency of *object sniffing,* with a significant reduction in duration in mice suspended for 3 weeks (U = 66.000, *p* = 0.0025) and in frequency in mice suspended for 1 week (F_(1,11)_ = 15.159, *p* = 0.0025), 2 weeks (F_(1,12)_ = 17.544, *p* = 0.0013) and 3 weeks (F_(1,15)_ = 25.394, *p* = 0.0001). Moreover, mice suspended for two weeks showed an increased latency in the first approach to the novel object (U = 35.000, *p* = 0.0455). A similar trend was observed after one and three weeks of suspension.

### 3.4. Brain NGF and BDNF Levels Were Not Significantly Different Among Groups

**Figure 8:**
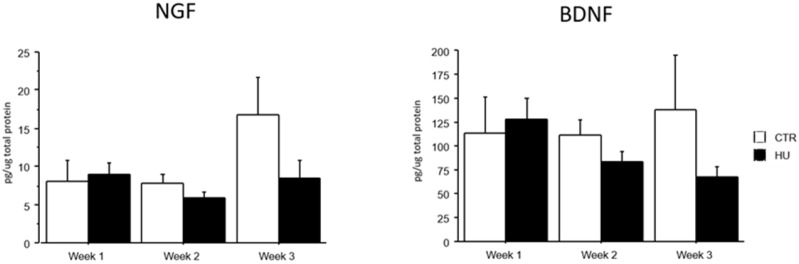
NGF and BDNF levels after suspension procedure. Data are shown as means ± S.E.M. CTR week1 = 6, CTR week2 = 7, CTR week3 = 7, HU week1 = 7, HU week2 = 7, HU week3 = 10.

The analysis of brain NGF and BDNF levels evidenced that all groups (week1, week2 and week3 in control and suspended subjects) did not significantly differ. However, a trend over the weeks towards a reduction in the levels of both neurotrophins was evident, only in suspended mice (Figure 8).

### 3.5. Some Behavioral Items During the First Day of Suspension Can Predict the Individual Response Profile During the Post Suspension Period

**Figure 9:**
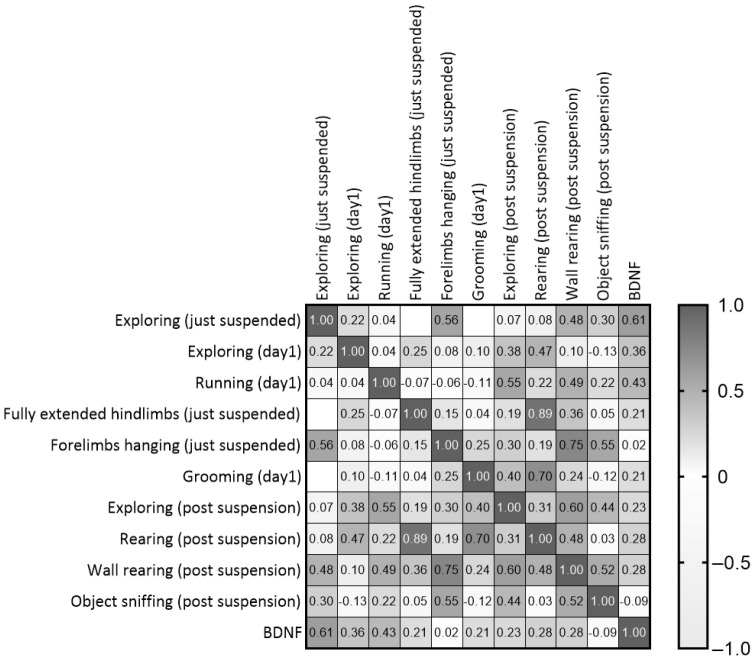
Correlation matrix between behaviors performed during the first day of suspension and the neurobehavioral outcomes revealed after the end of suspension.

Although the comparison between experimental groups revealed no differences in BDNF levels at the end of the suspension procedure, positive correlations were shown between post-suspension BDNF levels and behaviors performed during the suspension procedure. In particular, correlational analyses between behaviors performed during the suspension procedure and the novel object test revealed the following outcomes (Figure 9 and Table S2): *Exploring* in both just-suspended and day1 mice was positively correlated with *rearing* (r = 0.471, *p* = 0.03) and BDNF (r = 0.608, *p* = 0.0047); *running* during day1 was positively correlated with *Exploring* (r = 0.552, *p* = 0.0104); *fully extended hindlimbs in* just-suspended mice was positively correlated with *rearing* (r = 0.886, *p* = 0.0001); *grooming* during day1 was positively correlated with *rearing* (r = 0.703, *p* = 0.0003); and *forelimbs hanging* in just-suspended mice was positively correlated with *wall rearing* (r = 0.749, *p* = 0.0001) and *object sniffing* (r = 0.547, *p* = 0.0113).

### 3.6. BDNF, NGF and p75^NTR^ Transcripts in Optic Nerve Head After Suspension Procedure

**Figure 10:**
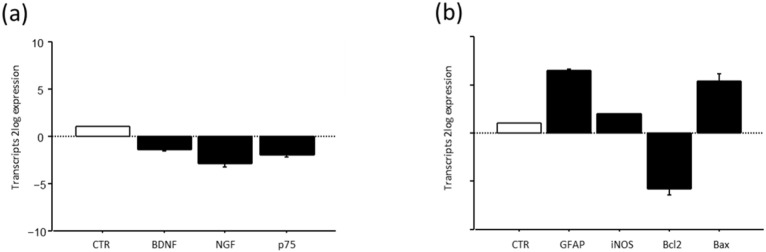
BDNF, NGF and their p75NTR pan-neurotrophin receptor (**a**) and GFAP. iNOS and Bcl2/Bax ratio (**b**) expression after suspension procedure. Data are shown as 2log expression (means ± S.E.M.), as calculated by the REST analysis equation. CTR indicates control values referred to as 1; HU.

A pilot study on the transcript expression of BDNF and NGF at week3 in the optic nerves of HU mice showed a significant deregulation of transcripts specific for both neurotrophins and the pan-receptor (Figure 10a). This deregulation was associated with a significant reduction in the Bcl2/Bax ratio (Figure 10a), associated with high GFAP and slight iNOS transcription (Figure 10b).

## 4. Discussion

The hindlimb unloading procedure strongly affects the behavioral profile of mice. In particular, a general decrease in explorative behaviors throughout the suspension weeks and a concomitant increase in *balancing* and *grooming* behaviors were clearly evident. Although the indexes of stress response complicate the interpretation of the behavioral results [35], in addition to a slight reduction in body weight in suspended mice, data indicated that suspended subjects showed a reduction in spontaneous activity and an increase in *grooming* behavior.

Hindlimb unloading markedly affects the behavioral profile of mice, leading to a robust reduction in spontaneous and exploratory activity and a concomitant increase in *grooming* and postural adjustment behaviors. The exposure duration emerged as the primary determinant of behavioral modulation, with most behaviors showing a clear temporal modulation, with higher exploratory activity at the onset of suspension followed by progressive habituation to the unloading condition. In particular, the elevated levels of exploratory behaviors—such as *exploration* and *head raising*—observed at the very beginning of the suspension, together with the subsequent increase in *fully extended hindlimbs* postures and the slight reduction in *forelimbs hanging* behaviors, may reflect an attempt to assess the environmental novelty and identify potential escape routes. This interpretation is supported by the tendency of animals to reach the cage walls, the suspension bar, and to move actively around the enclosure. These behaviors progressively subsided, whereas *running* and *grooming* became more frequent after the first day of suspension. This behavioral shift may indicate the emergence of displacement activities and/or, as previously stated, de-arousal processes associated with habituation to the stressful conditions induced by the suspension procedure [36]. These changes were accompanied by a slight reduction in body weight, indicating a general decrease in activity under suspension conditions. Sex differences were limited and behavior-specific, with females showing a transient increase in *running* behavior during the first week of suspension, suggesting that sex-related behavioral differences depend on the time window of observation rather than representing stable traits, likely reflecting distinct coping strategies. Behavioral changes under suspension are predominantly driven by exposure duration, highlighting a general process of habituation across behaviors, while sex modulates only specific behaviors and only at defined time points, underscoring the importance of longitudinal analyses in behavioral phenotyping.

The evaluation of behavioral profiles of the mice during the novel object test revealed that suspended mice explored the new cage less and rarely approached the novel object. It is worth mentioning that the experimental subjects were isolated during the suspension period. It is known that isolation per se and difficulty moving represent strong stressful conditions for rodents, being naturally social species [37]. Several behavioral studies reported that prolonged individual housing compromised the neurobehavioral status of mice, reducing their explorative attitude, enhancing anxiety-like behaviors and increasing behavioral responses to stress [38,39,40,41].

These effects are completely in line with the results obtained in the present study: suspended mice explored the cage for less time, rarely performed vertical movements and appeared less reactive to novelty. Moreover, although not significant and limited by the absence of stress marker analysis, we can speculate that the subtle tendency for a lack in NGF and BDNF increases observed in suspended mice across the three weeks could support this interpretation. Indeed, under chronic stressful conditions, the production of adrenal-glucocorticoids is exacerbated, leading to a reduction in NGF and BDNF levels and, subsequently, to a greater vulnerability to stress-related disorders [42,43,44]. This indicates that the gravity-related behavioral changes observed in animals that had undergone this procedure should be investigated very carefully. Although in the scientific community this model represents a recognized model to study the effects of simulated microgravity conditions on the musculoskeletal system, it should be taken into account that some changes in behavioral performance could also be correlated to the stressful conditions experienced by the animals, due to the isolation periods and strictly to the suspension methodology itself. Single-housing conditions can act as a significant confounding variable. In traditional hindlimb unloading (HU) protocols, prolonged social isolation can induce substantial alterations in stress physiology, influencing immune function and behavior in rodents, independently of the unloading procedure. Notably, social isolation has been demonstrated to modulate hypothalamic–pituitary–adrenal axis activity and adrenal responsiveness, which could mask or interact with HU-induced effects, resulting in outcomes that differ from those observed in socially housed animals [15]. Therefore, the social isolation and restraint embedded in HU paradigms can influence the behavioral profile of the animals, confounding the interpretations of these measures as specific consequences of simulated microgravitational conditions [41]. In this regard, it is important to mention that this test has long been and is still currently used to evaluate manipulations that are expected to affect depression-related behaviors [45]. The tail suspension test was, in fact, first introduced in 1985 [46] to measure the potential effectiveness of antidepressant drugs, and therefore neurobehavioral output should always be considered in the context of the adaptive response to chronic stress coupled with the effects of simulated microgravity exposure. This holds particular significance when assessing the impact of not only this experimental paradigm on learning or cognitive performance but also other physiological endpoints, since it is widely acknowledged that stress also has a significant impact on physical health, affecting the musculoskeletal, respiratory, cardiovascular, endocrine, gastrointestinal, and reproductive systems.

Tahimic and colleagues in 2019 developed a refinement of the traditional NASA single-housing HU methodology [47,48], where suspended mice were accommodated in pairs into each cage in order to reduce at least isolation stress [15]. Although both single and paired HU mice showed musculoskeletal deficits compared to the normally loaded controls, surprisingly, specific immune and stress-related responses were differentially affected by being in pairs. Therefore, the muscle and bone effects were basically related to gravitational changes, while neuro-immune-related effects appeared differently modulated by social environments [15,49].

In this context, the possibility to define some behavioral indicators of vulnerability to the suspension procedure represents an opportunity to select subjects less susceptible to the stress-related conditions involved in experimentations while improving animal welfare. The definition of an HU-specific ethogram clearly underlines the different trajectories in individual coping strategies during the suspension procedure and highlights possible predictive behavioral indicators of resilience to stress-related suspension. Indeed, present data show that active behavioral strategies during the first days of suspension are correlated with a less stressed behavioral profile after HU suspension regardless of the duration of the exposure.

Coherently with the known muscular impairments induced by HU, all suspended mice reduced vertical movements in the novel object test [50,51]. However, analysis across the weeks evidenced that several specific behaviors, such as *exploring*, *fully extended hindlimbs*, *forelimbs hanging* and *grooming*, are positively correlated with vertical movements after suspension, suggesting either the relevance of individual susceptibility in interpreting experimental data or that these behavioral items could be considered predictive behavioral indicators of resilience.

Furthermore, mice that were more explorative during the first two days performed more *rearing* and *wall rearing* behaviors after the end of the suspension. This could be explained by the motivational analogy underlying these behaviors: both *exploring* and vertical movements are related to the tendency to move around the cage or reach walls to increase the environmental input and be able to control and possibly escape from a predator [52]. *Rearing* behavior in fact exploits an upright posture, substantially ameliorating the capability of the subject to both sniff short- or even medium-distance olfactory cues and providing the possibility to visually score the environment even in the case of a minor obstacle. Such an exploratory activity is only displayed after the first phase of integration of environmental stimuli since it renders the subject more visible to potential predators; therefore, it is triggered when the environmental context is considered sufficiently safe, therefore signaling a lowered “anxiety” profile [52].

Interestingly, although the comparison between experimental groups revealed no differences in BDNF levels at the end of suspension procedure, the correlation between the post-suspension BDNF levels with behaviors performed during suspension revealed that just-suspended mice also showed higher levels of BDNF at the end of suspension, as well as a higher index of individual vulnerability to the suspension procedure, not related to the results derived from the comparison. In general, chronic stress could induce a reduction in BDNF expression in the brain due the crosstalk between glucocorticoid and neurotrophin systems [53,54]. In this case, although the evaluation of stress markers was not performed in this study, limiting the overall interpretation of the results, the positive correlation could be an index indicating that resilient subjects may perceive stress-related challenges differently with different molecular adaptive processes [55,56,57].

The predictive value of *grooming* behavior could be associated with the “cost” of this behavior during the suspension. To perform this behavior, the animal is required to bring the head towards the belly, performing an active behavior, showing a strong motivational boost and evidencing a possible resilient trait. This interpretation is also in line with the positive correlation found between the duration of *grooming* during the first week of suspension and the duration of post-suspension *rearing* behavior. Interestingly, early *running* behavior during suspension could predict a major explorative profile after the suspension procedure and could be considered another behavioral indicator of resilience. As for the explorative behaviors, the performance of *running* during the first days of suspension may be suggestive of an active coping strategy during suspension and at the same time a decreased sensitivity to stress conditions during and after the suspension.

To better characterize this model, we extended our attention to the optic nerve and the transcription of BDNF and NGF. The rationale behind the hypothesis is that the optic nerve transmits a series of unprocessed visual information from the eye to the brain, and due to this unconventional position, some neuro-ocular changes as well as edema might develop, influencing mice behavior [58]. Our analysis shows that BDNF and NGF as well as their pan-receptors were deregulated upon suspension, probably as a result of stressors released under this unconventional posture. This impaired expression of the main neurotrophins was associated with a significant decrease in the Bcl2/Bax ratio, indicative of the activation of the apoptotic pathway, and an increase in iNOS, implying the release of oxidative stress mediators [59]. At the same time, the high GFAP transcription would suggest the development of gliosis or the sustaining of local inflammation coupled to edema (optic neuritis) [58]. After all, GFAP is an intermediate cytoskeleton filament protein responsible for glia structure and mechanical strength, and therefore is a clear support to neighboring neurons [60]. Since this pilot study referred to optic nerves collected at week 3 (chronic process), it might be of interest to understand the NGF and BDNF paths in the acute phase. Otherwise, it might also be of interest to understand whether astrocyte reactivity can protect optic nerve function during the entire period of suspension, guaranteeing homeostasis [61].

The present data evidence that suspension affects the behavioral profile of mice, and behavioral analysis during suspension represents a valid tool to assess the susceptibility of mice to this procedure. However, it is worth mentioning that the interpretation of the behavioral data should take into account the different suspension methods employed. When considering animal welfare and the application of the 3R principles [62], the possibility to standardize such methods represents a fundamental issue in refining animal suffering and reducing the number of experimental subjects. The harmonization of the procedures represents a useful tool to collect scientific data comparable among research groups and avoid unnecessary replication [6,63].

### 4.1. Limitations and Future Perspectives

Although the scientific results reported in the present study could become instrumental to investigating individual behavioral reactions in ground-based models and refining methodological approaches to implement the translational value of the behavioral outcomes, it is essential to point out some methodological limitations that warrant cautious interpretation of the results. Firstly, the nature of stress, behavioral reactions and biomechanics of the process highlight the suitability of comparing HU and human weightlessness. Although comparison between hindlimb unloading and spaceflight animals revealed similar responses in many physiological systems (muscle, bone, heart, intestine, pulmonary, immune functions) [47], in the HU model, it is possible to only reproduce a partial unloading of the animal. Meanwhile, during spaceflight, the whole body is suspended. Additionally, the position and the isolation of the animal are totally unnatural, causing stress that could potentially confound the interpretations of the behavioral and biochemical results. Despite these cautions, hindlimb unloading is largely used to study simulated microgravity conditions because it allows for replicating muscle atrophy and bone demineralization in rodents, typical features associated with spaceflight experience. In this context, the analysis of the behavioral profile during the unloading period could become instrumental to identify critical windows of physiological or un-physiological adjustments to this condition and improve individual indicators of animal welfare. It is worth mentioning that in our study, the lack of a social housing experimental group makes it difficult to include a multivariate analysis to control for the impact of isolation. However, the sole presence of socially isolated experimental groups that differ for the period of suspension allows for discriminating the behavioral responses related to the time of suspension per se, the central aim of the present study. Moreover, since the differentiated levels of stress markers and neurotrophins in brain structures were not measured, which may be a limitation of our study design, we cannot exactly disentangle the mechanisms underlying behavioral stress-related responses to suspension procedures.

### 4.2. Conclusions

Overall, these findings suggest that specific behaviors in the exploratory domain are associated with a more adaptive behavioral trajectory during the suspension period, characterized by a rapid reduction in novelty-driven exploration and a stabilization of postural adjustments, indicative of greater behavioral flexibility and resilience to the unloading condition. Conversely, the progressive increase in postures such as *fully extended hindlimbs* or *grooming* behavior reflects a transition toward behavioral strategies supporting adaptation and coping under sustained unloading. In parallel, brain levels of NGF and BDNF were assessed in relation to behavioral outcomes. Although no significant group differences emerged, suspended mice exhibited a gradual reduction in both neurotrophins over time, paralleling the stabilization of behavioral responses and suggesting ongoing neuroplastic adaptations rather than acute stress effects. Exploratory analyses of the optic nerve further indicate that prolonged hindlimb unloading may induce neurotrophin dysregulation and glial-oxidative responses, suggesting that neuro-ocular pathways could represent an additional target of unloading-related stress and may contribute to long-term neural and behavioral adaptation under simulated microgravity conditions.

Future investigations including socially housed suspended animals, appropriate social housing controls, and a broader assessment of stress-related biomarkers will be critical to disentangle the specific physiological effects of unloading from the broader systemic impacts of isolation stress.

## Figures and Tables

**Table 1:** Post hoc pairwise comparisons for behavioral duration and frequency, obtained from linear mixed-effects models. Comparisons are reported only for statistically significant contrasts (Tukey-adjusted *p* values). Results are shown across suspension periods (just suspended, day1, week1, week2, week3). Estimates represent differences between levels, with associated standard errors (SEs), degrees of freedom (dfs), *t* values, and adjusted *p* values.

Measure	Behavior	Contrast	Estimate	SE	df	t Value	*p* Value
Duration	Exploring	just suspended–day1	83.54	19.3	21.6	4.321	0.0024
Duration	Exploring	just suspended–week1	95.01	18.8	20.6	5.057	0.0005
Duration	Exploring	just suspended–week2	100.82	18.8	20.6	5.366	0.0002
Duration	Exploring	just suspended–week3	97.32	19.3	21.6	5.034	0.0004
Duration	Running	just suspended–week1	−75.9	16.7	24.2	−4.554	0.0011
Duration	Running	day1–week3	55.2	16.2	22.8	3.403	0.0188
Duration	Running	week1–week2	58.2	16.2	22.8	3.583	0.0124
Duration	Running	week1–week3	86.6	16.5	23.4	5.245	0.0002
Duration	Fully extended hindlimbs	just suspended–week1	−22.69	6.44	25.4	−3.523	0.013
Duration	Vertical head raising	just suspended–day1	23.32	7.58	24.9	3.074	0.037
Duration	Vertical head raising	just suspended–week1	27.56	7.4	24	3.724	0.0085
Duration	Vertical head raising	just suspended–week2	38.58	7.4	24	5.212	0.0002
Duration	Vertical head raising	just suspended–week3	44.47	7.58	24.9	5.862	0.0001
Duration	Forelimbs hanging	just suspended–week1	12.75	3.57	25.4	3.573	0.0115
Duration	Forelimbs hanging	just suspended–week2	15.3	3.57	25.4	4.286	0.002
Duration	Forelimbs hanging	just suspended–week3	17.44	3.61	26.4	4.83	0.0005
Duration	Grooming	just suspended–week1	−64.95	19.1	24.9	−3.393	0.018
Frequency	Exploring	just suspended–day1	24.178	4.84	25.7	4.997	0.0003
Frequency	Exploring	just suspended–week1	29.099	4.77	24.5	6.102	0.0001
Frequency	Exploring	just suspended–week2	33.599	4.77	24.5	7.045	0.0001
Frequency	Exploring	just suspended–week3	33.303	4.84	25.7	6.883	0.0001
Frequency	Running	week1–week3	15.969	2.4	23.4	6.649	0.0001
Frequency	Fully extended hindlimbs	just suspended–week1	−9.253	1.82	25.3	−5.094	0.0003
Frequency	Fully extended hindlimbs	week1–week3	7.376	1.8	24.6	4.089	0.0034
Frequency	Alternative extended hindlimbs	just suspended–week2	6.471	2.01	21.4	3.225	0.0293
Frequency	Vertical head raising	just suspended–week3	19.4	3.45	24.2	5.625	0.0001
Frequency	Forelimbs hanging	just suspended–week3	8.7	1.6	26.7	5.444	0.0001
Frequency	Grooming	just suspended–week1	−13.674	3.7	24.3	−3.693	0.009

## Data Availability

Data will be made available on request.

## Supplementary Materials

The following supporting information can be downloaded at: https://www.mdpi.com/article/10.3390/life16010137/s1, Figure S1: Sex-related differences in balancing behaviors during suspension; Table S1: Post hoc pairwise comparisons derived from linear mixed-effects models (LMMs) evaluating the effects of suspension period across weeks separately in female and male mice; Table S2: Specific values of the correlation matrix between behaviors performed during the first day of suspension and the neurobehavioral outcomes collected after the end of suspension.
