# Supplementary material for: Behavioral and Neurobiological Correlates of Resilience in the Hindlimb Unloading Mouse Model: A Longitudinal Ethogram with Neurotrophin Profile
Source: Life (Basel). 2026 Jan 15;16(1):137. doi: 10.3390/life16010137 (PMC12842622; doi:10.3390/life16010137)
Supplement: Supplementary file 1 [file life-16-00137-s001.zip › life-4057089-supplementary.pdf]

life

MDPI

Supplementary materials

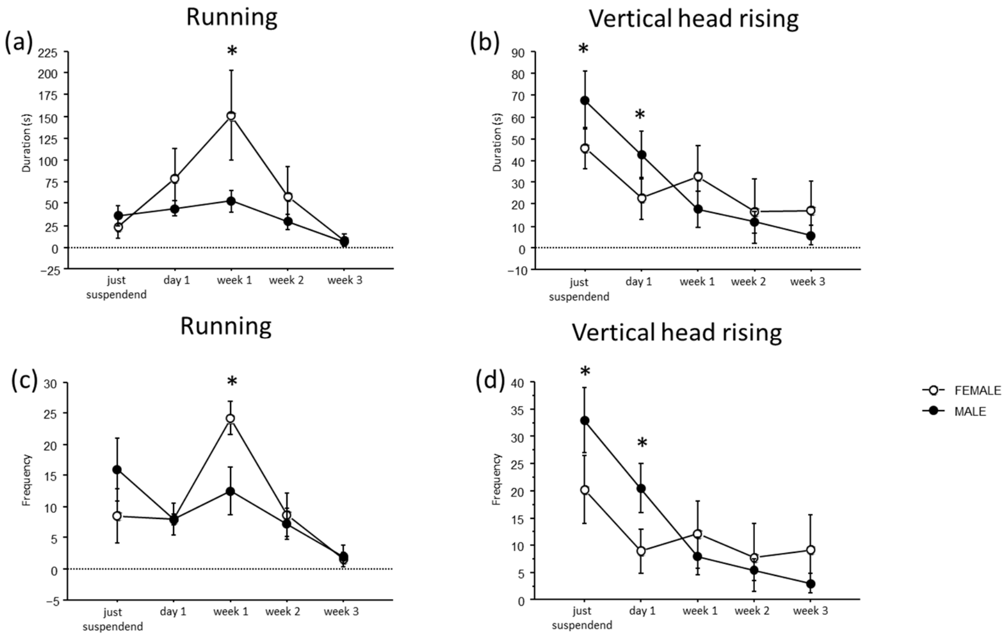

Figure S1. Sex-related differences in balancing behaviors during suspension. An increase in the duration and frequency of *Running* (a,c) or *Vertical head rising* (**b**,**d**) has been observed in females. Data are shown as estimated marginal means from a linear mixed-effects model with period of suspension, sex, and their interaction as fixed effects and subject as a random effect. Estimates are based on Tukey-adjusted pairwise comparisons, with degrees of freedom computed using the Satterthwaite method (see Table S1); Females = 4, Males = 4.

Table S1. Post hoc pairwise comparisons derived from linear mixed-effects models (LMMs) evaluating the effects of suspension periodacross weeks separately in female and male mice. Analyses were conducted for both behavioral duration and frequency measures. Contrasts report estimated differences between time points (Just Suspended, day1, week1, week2, and week3 of suspension), with corresponding standard errors (SE), degrees of freedom (df), t-ratios, and Tukey-adjusted p-values. Only contrasts relevant to sex-specific temporal effects are shown. Statistical significance was set at p < 0.05.

Measure	Behavior	Sex	Contrast	Estimate	SE	df	t-ratio	p-value
Duration	Running	Female	just suspended vs day1	-80.69	25.3	26.9	-3193	0.027
Duration	Running	Female	just suspended vs week1	-135.14	24.2	25.4	-5.59	<0.0001
Duration	Running	Female	1day vs week3	71.54	23	22.8	3.117	0.0355
Duration	Running	Male	just suspended vs week3	30.58	23	22.8	1.332	0.6747
Duration	Head Raising	Male	just suspended vs week1	50.28	10.2	22.6	4.938	0.0005

Duration	Head Raising	Male	just suspended vs week2	56.15	10.2	22.6	5.514	0.0001
Duration	Head Raising	Male	just suspended vs week3	62.02	10.2	22.6	6.091	<0.0001
Frequency	Running	Female	just suspended vs week1	-15.56	3.49	25.5	-4.459	0.0013
Frequency	Running	Female	week1 vs week3	21.44	3.45	24	6.218	<0.0001
Frequency	Running	Male	just suspended vs week3	14	3.34	22.7	4.186	0.003
Frequency	Hanging	Female	just suspended vs week1	7.535	2.26	26.7	3.336	0.0194
Frequency	Hanging	Female	just suspended vs week2	8.66	2.26	26.7	3.834	0.0058
Frequency	Hanging	Male	just suspended vs week3	8.5	2.22	24.6	3.834	0.0063

Table S2. Specific values of the correlation matrix between behaviors performed during the first day of suspension and the neurobehavioral outcomes collected after the end of suspension.

During early suspension	Post suspension data	r	p	IC 95%
Exploring (day1)	Rearing	0,471	0,03	0,036-0,756
Exploring (just suspended)	BDNF	0,608	0,0047	0,213-0,832
Running (day1)	*Exploring*	0,552	0,0104	0,145-0,799
Fully extended hindlimbs (just suspended)	Rearing	0,886	0,0001	0,729-0,954
Grooming (day1)	Rearing	0,703	0,0003	0,379-0,874
Forelimbs hanging (just suspended)	Wall rearing	0,749	0,0001	0,459-0,895
Forelimbs hanging (just suspended)	Object sniffing	0,547	0,0113	0,138-0,797
